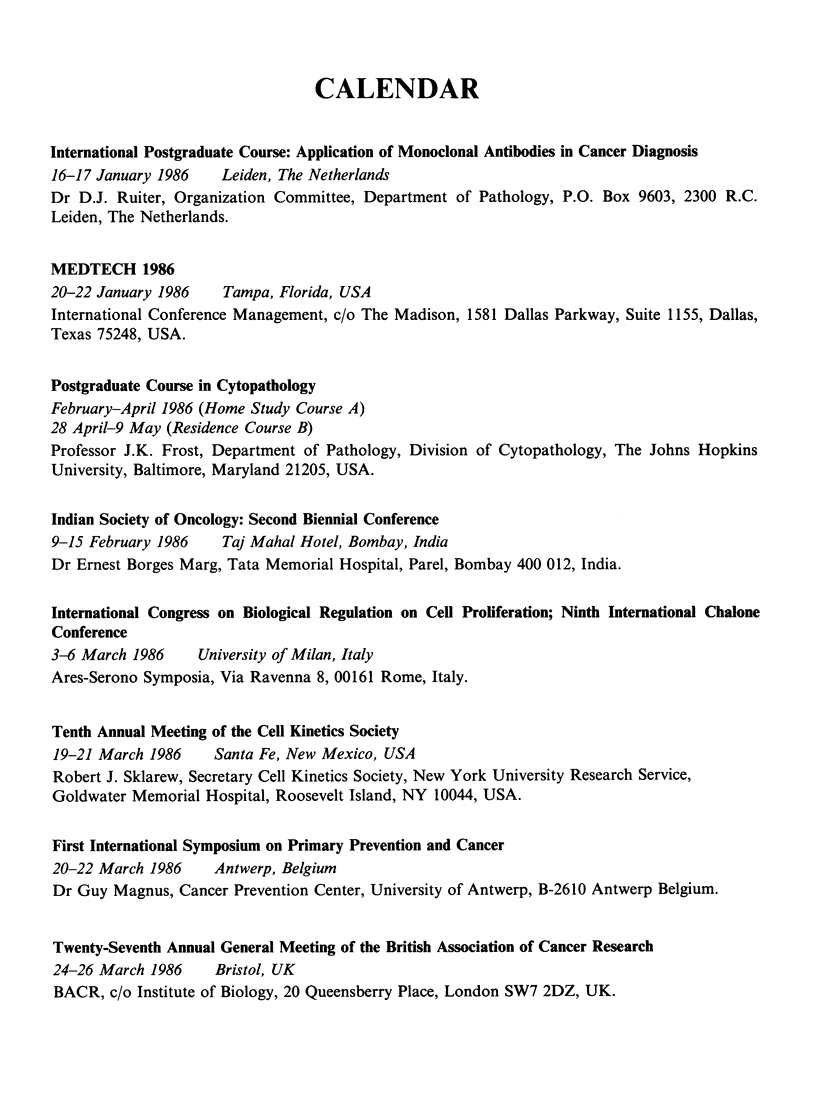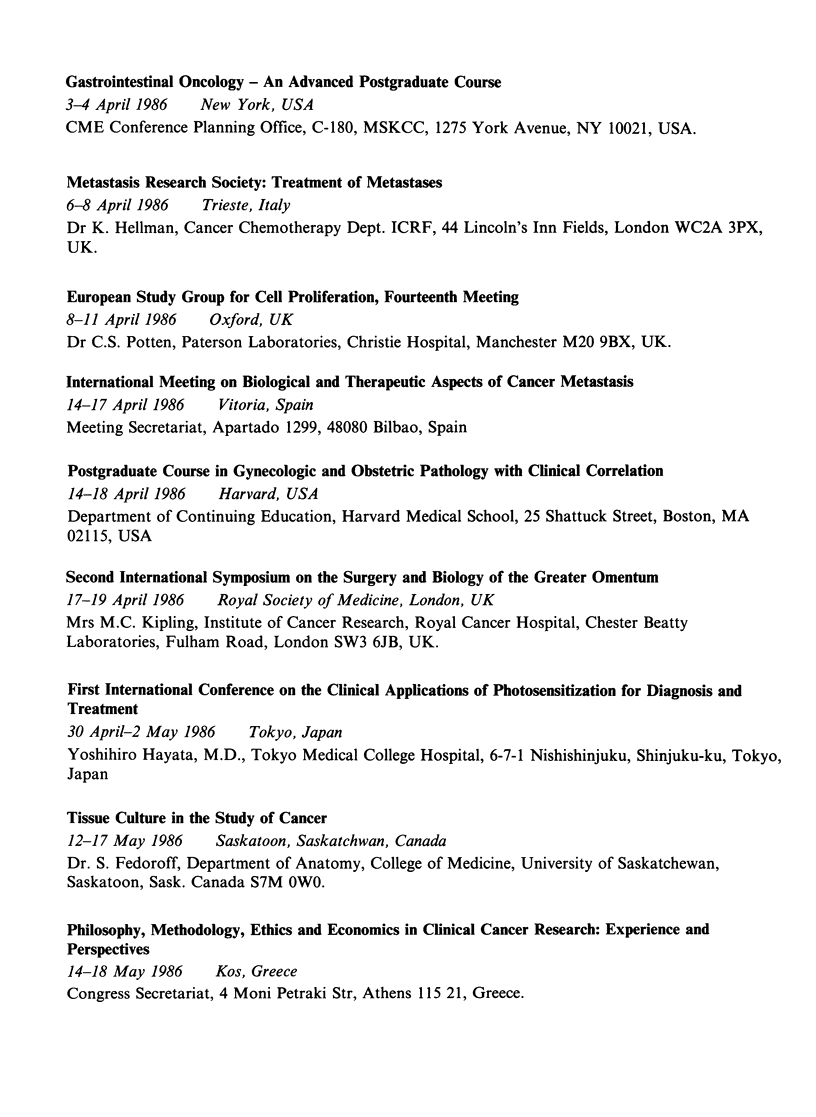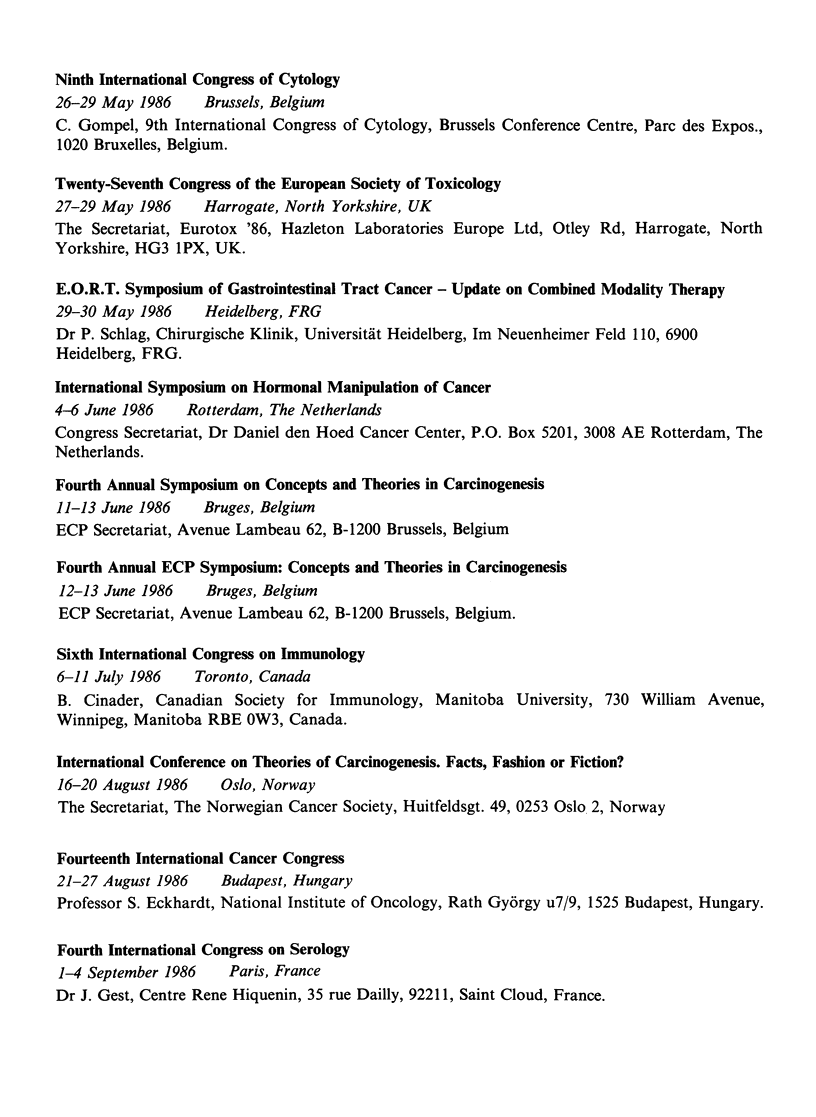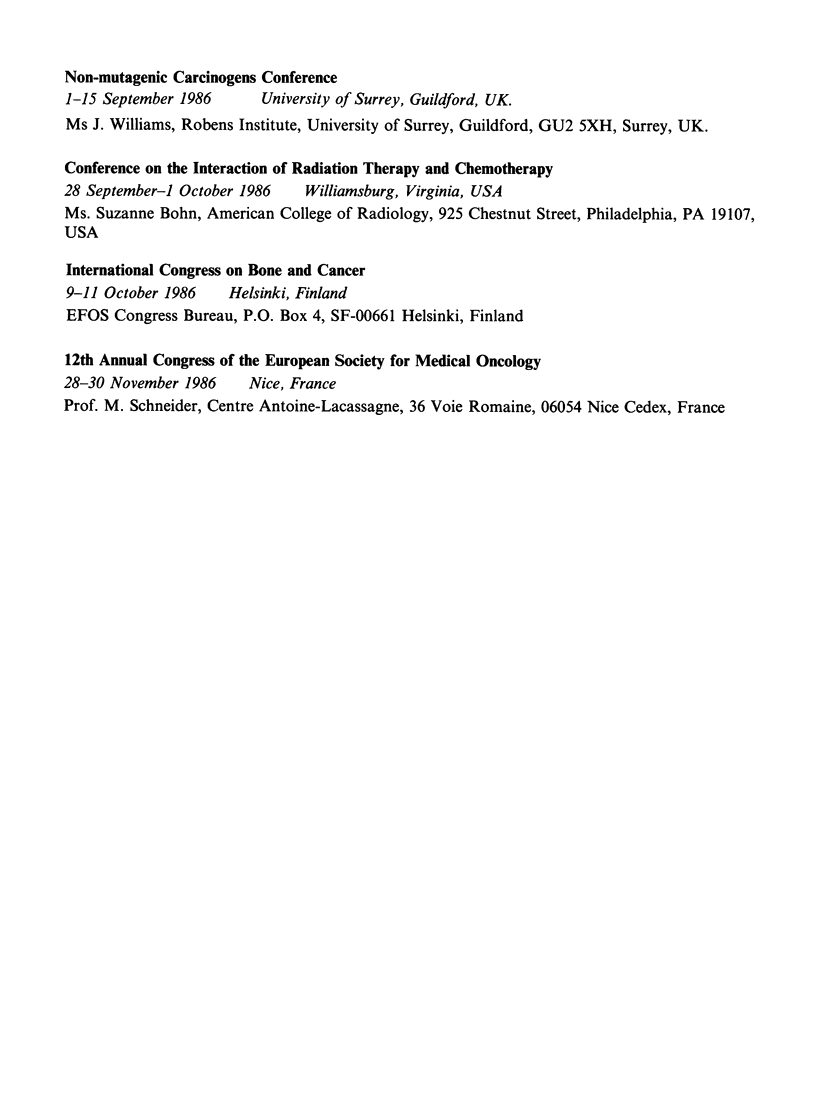# Calendar

**Published:** 1986-01

**Authors:** 


### CALENDAR

**International Postgraduate Course: Application of Monoclonal Antibodies in Cancer Diagnosis**
*16–17 January 1986 Leiden, The Netherlands*
Dr D.J. Ruiter, Organization Committee, Department of Pathology, P.O. Box 9603, 2300 R.C. Leiden, The Netherlands.

**MEDTECH 1986**
*20–22 January 1986 Tampa, Florida, USA*
International Conference Management, c/o The Madison, 1581 Dallas Parkway, Suite 1155, Dallas, Texas 75248, USA.

**Postgraduate Course in Cytopathology**
*February–April 1986 (Home Study Course A)*
*28 April–9 May (Residence Course B)*
Professor J.K. Frost, Department of Pathology, Division of Cytopathology, The Johns Hopkins University, Baltimore, Maryland 21205, USA.

**Indian Society of Oncology: Second Biennial Conference**
*9–15 February 1986 Taj Mahal Hotel, Bombay, India*
Dr Ernest Borges Marg, Tata Memorial Hospital, Parel, Bombay 400 012, India.

**International Congress on Biological Regulation on Cell Proliferation; Ninth International Chalone Conference**
*3–6 March 1986 University of Milan, Italy*
Ares-Serono Symposia, Via Ravenna 8, 00161 Rome, Italy.

**Tenth Annual Meeting of the Cell Kinetics Society**
*19–21 March 1986 Santa Fe, New Mexico, USA*
Robert J. Sklarew, Secretary Cell Kinetics Society, New York University Research Service, Goldwater Memorial Hospital, Roosevelt Island, NY 10044, USA.

**First International Symposium on Primary Prevention and Cancer**
*20–22 March 1986 Antwerp, Belgium*
Dr Guy Magnus, Cancer Prevention Center, University of Antwerp, B-2610 Antwerp Belgium.

**Twenty-Seventh Annual General Meeting of the British Association of Cancer Research**
*24–26 March 1986 Bristol, UK*
BACR, c/o Institute of Biology, 20 Queensberry Place, London SW7 2DZ, UK.

**Gastrointestinal Oncology – An Advanced Postgraduate Course**

*3–4 April 1986* *New York, USA*

CME Conference Planning Office, C-180, MSKCC, 1275 York Avenue, NY 10021, USA.

**Metastasis Research Society: Treatment of Metastases**

*6–8 April 1986* *Trieste, Italy*

Dr K. Hellman, Cancer Chemotherapy Dept. ICRF, 44 Lincoln's Inn Fields, London WC2A 3PX, UK.

**European Study Group for Cell Proliferation, Fourteenth Meeting**

*8–11 April 1986* *Oxford, UK*

Dr C.S. Potten, Paterson Laboratories, Christie Hospital, Manchester M20 9BX, UK.

**International Meeting on Biological and Therapeutic Aspects of Cancer Metastasis**

*14–17 April 1986* *Vitoria, Spain*

Meeting Secretariat, Apartado 1299, 48080 Bilbao, Spain

**Postgraduate Course in Gynecologic and Obstetric Pathology with Clinical Correlation**

*14–18 April 1986* *Harvard, USA*

Department of Continuing Education, Harvard Medical School, 25 Shattuck Street, Boston, MA 02115, USA

**Second International Symposium on the Surgery and Biology of the Greater Omentum**

*17–19 April 1986* *Royal Society of Medicine, London, UK*

Mrs M.C. Kipling, Institute of Cancer Research, Royal Cancer Hospital, Chester Beatty Laboratories, Fulham Road, London SW3 6JB, UK.

**First International Conference on the Clinical Applications of Photosensitization for Diagnosis and Treatment**

*30 April–2 May 1986* *Tokyo, Japan*

Yoshihiro Hayata, M.D., Tokyo Medical College Hospital, 6-7-1 Nishishinjuku, Shinjuku-ku, Tokyo, Japan

**Tissue Culture in the Study of Cancer**

*12–17 May 1986* *Saskatoon, Saskatchwan, Canada*

Dr. S. Fedoroff, Department of Anatomy, College of Medicine, University of Saskatchewan, Saskatoon, Sask. Canada S7M 0W0.

**Philosophy, Methodology, Ethics and Economics in Clinical Cancer Research: Experience and Perspectives**

*14–18 May 1986* *Kos, Greece*

Congress Secretariat, 4 Moni Petraki Str, Athens 115 21, Greece.

**Ninth International Congress of Cytology**

*26–29 May 1986 Brussels, Belgium*

C. Gompel, 9th International Congress of Cytology, Brussels Conference Centre, Parc des Expos., 1020 Bruxelles, Belgium.

**Twenty-Seventh Congress of the European Society of Toxicology**

*27–29 May 1986 Harrogate, North Yorkshire, UK*

The Secretariat, Eurotox '86, Hazleton Laboratories Europe Ltd, Otley Rd, Harrogate, North Yorkshire, HG3 1PX, UK.

**E.O.R.T. Symposium of Gastrointestinal Tract Cancer – Update on Combined Modality Therapy**

*29–30 May 1986 Heidelberg, FRG*

Dr P. Schlag, Chirurgische Klinik, Universität Heidelberg, Im Neuenheimer Feld 110, 6900 Heidelberg, FRG.

**International Symposium on Hormonal Manipulation of Cancer**

*4–6 June 1986 Rotterdam, The Netherlands*

Congress Secretariat, Dr Daniel den Hoed Cancer Center, P.O. Box 5201, 3008 AE Rotterdam, The Netherlands.

**Fourth Annual Symposium on Concepts and Theories in Carcinogenesis**

*11–13 June 1986 Bruges, Belgium*

ECP Secretariat, Avenue Lambeau 62, B-1200 Brussels, Belgium

**Fourth Annual ECP Symposium: Concepts and Theories in Carcinogenesis**

*12–13 June 1986 Bruges, Belgium*

ECP Secretariat, Avenue Lambeau 62, B-1200 Brussels, Belgium.

**Sixth International Congress on Immunology**

*6–11 July 1986 Toronto, Canada*

B. Cinader, Canadian Society for Immunology, Manitoba University, 730 William Avenue, Winnipeg, Manitoba RBE 0W3, Canada.

**International Conference on Theories of Carcinogenesis. Facts, Fashion or Fiction?**

*16–20 August 1986 Oslo, Norway*

The Secretariat, The Norwegian Cancer Society, Huitfeldsgt. 49, 0253 Oslo 2, Norway

**Fourteenth International Cancer Congress**

*21–27 August 1986 Budapest, Hungary*

Professor S. Eckhardt, National Institute of Oncology, Rath György u7/9, 1525 Budapest, Hungary.

**Fourth International Congress on Serology**

*1–4 September 1986 Paris, France*

Dr J. Gest, Centre Rene Hiquenin, 35 rue Dailly, 92211, Saint Cloud, France.

**Non-mutagenic Carcinogens Conference**

*1–15 September 1986* *University of Surrey, Guildford, UK.*

Ms J. Williams, Robens Institute, University of Surrey, Guildford, GU2 5XH, Surrey, UK.

**Conference on the Interaction of Radiation Therapy and Chemotherapy**

*28 September–1 October 1986* *Williamsburg, Virginia, USA*

Ms. Suzanne Bohn, American College of Radiology, 925 Chestnut Street, Philadelphia, PA 19107, USA

**International Congress on Bone and Cancer**

*9–11 October 1986* *Helsinki, Finland*

EFOS Congress Bureau, P.O. Box 4, SF-00661 Helsinki, Finland

**12th Annual Congress of the European Society for Medical Oncology**

*28–30 November 1986* *Nice, France*

Prof. M. Schneider, Centre Antoine-Lacassagne, 36 Voie Romaine, 06054 Nice Cedex, France